# Maternal Risk of Cardiovascular Disease After Use of Assisted Reproductive Technologies

**DOI:** 10.1001/jamacardio.2023.2324

**Published:** 2023-08-09

**Authors:** Maria C. Magnus, Abigail Fraser, Siri E. Håberg, Kristiina Rönö, Liv Bente Romundstad, Christina Bergh, Anne Lærke Spangmose, Anja Pinborg, Mika Gissler, Ulla-Britt Wennerholm, Bjørn Olav Åsvold, Deborah A. Lawlor, Signe Opdahl

**Affiliations:** 1 Centre for Fertility and Health, Norwegian Institute of Public Health, Oslo, Norway; 2 Population Health Sciences, Bristol Medical School, Bristol, United Kingdom; 3 MRC Integrative Epidemiology Unit at the University of Bristol, Bristol, United Kingdom; 4 Department of Obstetrics and Gynecology, University of Helsinki and Helsinki University Hospital, Helsinki, Finland; 5 Spiren Fertility Clinic, Trondheim, Norway; 6 Department of Obstetrics and Gynecology, Institute of Clinical Sciences, Sahlgrenska Academy, University of Gothenburg and Region Västra Götaland, Sahlgrenska University Hospital, Gothenburg, Sweden; 7 Fertility Department, Copenhagen University Hospital, Rigshospitalet, Denmark, Copenhagen; 8 Finnish Institute for Health and Welfare, Helsinki, Finland, Region Stockholm, Academic Primary Health Care Centre, Stockholm, Sweden; 9 Karolinska Institutet, Department of Molecular Medicine and Surgery, Stockholm, Sweden; 10 K. G. Jebsen Center for Genetic Epidemiology, Norwegian University of Science and Technology, Trondheim, Norway; 11 Department of Public Health and Nursing, Norwegian University of Science and Technology, Trondheim, Norway; 12 Department of Endocrinology, Clinic of Medicine, St Olavs Hospital, Trondheim University 1 Hospital, Trondheim, Norway

## Abstract

**Importance:**

The use of assisted reproductive technologies (ARTs) is steadily increasing worldwide. The outcomes associated with treatment for an individual’s long-term health, including risk of cardiovascular disease (CVD), remain largely unknown, due to the small number of studies and their limited follow-up time.

**Objective:**

To study whether the risk of CVD is increased among individuals who have given birth after ART compared with those who have given birth without ART.

**Design, Setting, and Participants:**

A registry-based cohort study was conducted using nationwide data from Denmark (1994-2014), Finland (1990-2014), Norway (1984-2015), and Sweden (1985-2015). Data analysis was conducted from January to August 2022. A total of 2 496 441 individuals with a registered delivery in the national birth registries during the study period were included, and 97 474 (4%) of these gave birth after ART.

**Exposures:**

Data on ART conception were available from ART quality registries and/or medical birth registries.

**Main Outcomes and Measures:**

Information on CVD was available from patient and cause of death registries. The risk of CVD was estimated with Cox proportional hazards regression, adjusting for age, calendar year of start of follow-up, parity, diagnosis of polycystic ovary syndrome, diabetes, chronic hypertension, and country.

**Results:**

Median follow-up was 11 (IQR, 5-18) years. The mean (SD) age of women with no use of ART was 29.1 (4.9) years, and the age of those who used ART was 33.8 (4.7) years. The rate of any CVD was 153 per 100 000 person-years. Individuals who gave birth after using ART had no increased risk of CVD (adjusted hazard ratio [AHR], 0.97; 95% CI, 0.91-1.02), with evidence of heterogeneity between the countries (*I*^2^ = 76%; *P* = .01 for heterogeneity). No significant differences in the risk of ischemic heart disease, cerebrovascular disease, stroke, cardiomyopathy, heart failure, pulmonary embolism, or deep vein thrombosis were noted with use of ART. However, there was a tendency for a modest reduction in the risk of myocardial infarction (AHR, 0.80; 95% CI, 0.65-0.99), with no notable heterogeneity between countries.

**Conclusions and Relevance:**

The findings of this study suggest that women who gave birth after ART were not at increased risk of CVD over a median follow-up of 11 years compared with those who conceived without ART. Longer-term studies are needed to further examine whether ART is associated with higher risk of CVD.

## Key Points

**Question:**

Is use of assisted reproductive technologies (ART) associated with increased risk of cardiovascular disease (CVD) among parous individuals?

**Findings:**

In this cohort study, among 2 496 441 parous individuals in Denmark, Finland, Norway, and Sweden, those who gave birth after ART were not at increased risk of later CVD compared with individuals who conceived without ART when adjusting for other CVD risk factors. No significant differences in the risk of ischemic heart disease, cerebrovascular disease, stroke, cardiomyopathy, heart failure, pulmonary embolism, or deep vein thrombosis were noted.

**Meaning:**

Most individuals who have used ART are still young, and this study found no association between use of ART and a higher risk of CVD; studies with longer follow-up are needed.

## Teaser

This cohort study assesses the association between use of assisted reproductive technologies and risk of cardiovascular disease among parous women.

## Introduction

The use of assisted reproductive technologies (ARTs) is steadily increasing.^1,2^ There are several potential explanations for how use of ARTs could increase cardiovascular disease (CVD). Assisted reproductive technology itself might increase the risk of CVD, for example, through ovarian hyperstimulation contributing to a prothrombotic state and endothelial injury.^3,4,5^ Individuals undergoing ART may also have accelerated biological aging, as measured by DNA methylation levels, which might influence their risk of chronic diseases.^6^ Conditions known to contribute to infertility, and therefore the use of ART, such as polycystic ovary syndrome and endometriosis, are also linked to a higher risk of CVD.^7,8^ In addition, any increased risk of CVD among individuals who have used ART may be explained by other confounding mechanisms or reverse causality (ie, preexisting undiagnosed CVD influencing infertility).

A meta-analysis summarized the current evidence on risk of CVD in individuals who delivered after using ART.^9^ The authors identified 6 observational studies that included 41 910 parous women who had used ART and 1 400 202 who had not and concluded that there was weak statistical evidence of a higher risk of cerebrovascular disease among individuals who had used ART (combined hazard ratio [HR], 1.25; 95% CI, 0.96-1.63) and no evidence of a higher risk of ischemic heart disease (combined HR, 0.91; 95% CI, 0.67-1.25).^9^ The authors stated that the small number of events (the exact number of events was not reported), the heterogeneity between the studies, and the short follow-up time did not permit any firm conclusions.

The aim of the current study was to assess the association between use of ART and risk of CVD among parous women. In comparison with the meta-analysis,^9^ by using a large Nordic registry linkage study, we have a considerably larger number of participants (total N = 2 496 441, with 97 474 having used ART), and were able to adjust for preexisting CVD risk factors.

## Methods

### Committee of Nordic ART and Safety

This study included all individuals in a Nordic registry linkage as part of the Committee of Nordic ART and Safety Collaboration.^10^ The linkage includes data on all deliveries registered in the medical birth registries in Denmark from 1994 to 2014, Finland from 1990 to 2014, Norway from 1984 to 2015, and Sweden from 1985 to 2015, depending on when the countries started registration of ART treatment (total 4 149 279 parous women). Follow-up information on hospital contact and death from CVD among these individuals was available through national patient and cause of death registries. The start of follow-up in the patient registries was January 1, 1987, for Sweden and Finland; January 1, 1994, for Denmark; and January 1, 2008, for Norway. We followed up individuals from 2 years after their first registered delivery after the start of the national patient registry. We defined the index delivery as the first delivery after the start of follow-up. The end of follow-up was December 31, 2015, for Norway and Sweden, and December 31, 2014, for Denmark and Finland. This study was approved by regional ethical committees in Norway and Sweden. In Denmark and Finland, ethical approval is not required for medical research solely based on registry data. This study is reported according to the Strengthening the Reporting of Observational Studies in Epidemiology (STROBE) reporting guideline.

### Assisted Reproductive Technologies

We defined each registered delivery as conceived with or without the use of ART based on information provided in the national birth registries for Norway and Finland and in national ART registries for Denmark and Sweden.^10^ We were not able to identify whether a delivery was the result of non-ART medically assisted reproduction, such as intrauterine insemination or individuals only using medications for ovulation induction. The main exposure was any delivery after using ART, and individuals were classified as exposed from the time of their first delivery from use of ART (yes vs no). Secondary exposures included fertilization method (in vitro fertilization [IVF] with or without intracytoplasmic sperm injection [ICSI]), or the use of fresh vs frozen embryo transfer, compared with individuals who had not used ART. These terms related to ART are further defined in eTable 1 in Supplement 1.

### Cardiovascular Disease

The main outcome was CVD defined as any registration of ischemic heart disease (including myocardial infarction), cerebrovascular disease (including stroke), cardiomyopathy, heart failure, pulmonary embolism, and deep vein thrombosis. We also evaluated each of these subcategories of CVD separately. Diagnoses in the patient and cause of death registries are coded according to the *International Classification of Diseases, 8th Revision* (*ICD-8*), *International Classification of Diseases, 9th Revision* (*ICD-9*), and *International Statistical Classification of Diseases and Related Health Problems, 10th Revision* (*ICD-10*). The *ICD* codes used to define CVD are available in eTable 2 in Supplement 1. In Denmark, diagnoses recorded on outpatient visits in specialized health care units have been included since 1995 in public hospitals and since 2003 in private clinics. In Finland, outpatient visits in specialized health care units in public hospitals have been included since 1998. Outpatient visits in specialized health care units in both public hospitals and private clinics have been included since 2008 in Norway and since 2001 in Sweden.

### Covariates

Potential confounders included background characteristics that could be associated with both the likelihood of using ART and the risk of CVD. From the delivery record of the index delivery, information on maternal age (continuous), parity (number of deliveries before the start of follow-up: 0, 1, 2, and ≥3), chronic hypertension present before delivery (yes vs no), prepregnancy or first trimester body mass index (underweight [<18.5], normal weight [18.5-24.9], overweight [25.0-29.9], and obese [≥30.0], calculated as weight in kilograms divided by meters squared), and tobacco use (yes vs no) was available from the birth registries. We had information on tobacco use/body mass index only for a subgroup of participants, as this information was introduced at varying time points in the birth registries (1994/2004 Denmark, 1990/2004 Finland, 1982/1982 Sweden, and 1999/2007 Norway). Highest achieved educational level (primary/secondary, short tertiary/bachelor, long tertiary/master, or higher) was available from national education databases in Denmark, Finland and Sweden, but not from Norway. Information on diabetes (yes vs no; *ICD-8* and *ICD-9* code 250, and *ICD-10* codes E10-14) and polycystic ovary syndrome (yes vs no; *ICD-8* code 256.9, *ICD-9* code 2654, and *ICD-10* code E28.2) at the start of follow-up was obtained from the national patient registries. We also evaluated multiple gestation and pregnancy complications obtained from the birth records as potential mediators, including preterm birth (birth <37 completed weeks; yes vs no), hypertensive disorders of pregnancy (yes vs no), stillbirth (yes vs no) and small for gestational age (birthweight <22% of expected mean; yes vs no). Hypertensive disorders in pregnancy were registered in the Danish and Finnish national patient registries and the Norwegian, Finnish, and Swedish medical birth registries according to national adaptations of the *International Statistical Classifications of Diseases and Related Health Problems*.^11^ The definition of small for gestational-age was based on Marsál equations,^12^ where 22% below the mean has been the clinically used in the Nordic countries for a large part of the study period.^13^ This corresponds approximately to the 2.3 percentile of a intrauterine growth standard. Marsál et al estimated expected birthweight according to gestational age from intrauterine (ultrasonography) measurements of ongoing, presumably healthy pregnancies in Sweden and Denmark.

### Statistical Analysis

Data analysis was conducted from January to August 2022. We used Cox proportional hazards regression to estimate the association between delivery after ART and risk of CVD. Individuals who delivered were the unit of analysis, and they could contribute both unexposed and exposed follow-up time, where individuals were considered exposed from their first delivery after using ART. We analyzed data from all countries combined, and subsequently conducted separate analyses for each country. The start of follow-up for each woman in the analysis was 2 years after their first delivery. Women were followed up until the first registration of a CVD diagnosis, emigration (not available for Finland), death from other causes, or the end of follow-up. The main multivariable model adjusted for confounders, including age, parity, diabetes, chronic hypertension, polycystic ovary syndrome, calendar year of start of follow-up, and country. A separate multivariable model evaluated the role of multiple gestation and pregnancy complications (preterm birth, hypertensive disorders of pregnancy, small for gestational age, and stillbirth) as potential mediators of the association between delivery by ART and CVD risk, which were entered as time-varying covariates. The assumption of proportional hazards was assessed by visually inspecting the Schoenfeld residuals. We assessed differences in the estimates between the countries using the *I*^2^ heterogeneity test.

We explored 2 separate sensitivity analyses in which we evaluated the adjustment for additional covariates only available for subsamples of the study population. First, we adjusted for tobacco use and body mass index measured at index delivery for the 50% of the study population across the 4 countries for whom this information was available. Second, we explored additional adjustment for educational level among the 93% of the total study population from Denmark, Finland, and Sweden with this information available.

We conducted additional sensitivity analyses to evaluate the robustness of our findings. We excluded pulmonary embolism and deep vein thrombosis from the outcome definition. We conducted an analysis also including women who were parous at the start of follow-up. We conducted a stratified analysis by year at start of follow-up, categorized as 1988-1997, 1998-2007, and 2008-2017. This stratified analysis was conducted to evaluate the potential role of changes in ART procedures over the long follow-up period.

Secondary exposures included fertilization method (IVF with or without ICSI) and the use of fresh vs frozen embryo transfer, compared with individuals who had not used ART. If similar associations would be observed for use of IVF with and without ICSI, compared with individuals who did not use ART, then this may strengthen the hypothesis that the association reflects a role of the procedure and not underlying fertility in the individual, as ICSI is primarily used for male fertility problems in the Nordic countries.^2^ Previous studies indicate some difference in the risk of hypertensive disorders of pregnancy according to fresh vs frozen embryo transfer, and that fresh embryo results in increased risk of small for gestational age, whereas frozen embryo increases risk of large for gestational age,^14^ which highlights the importance of clarifying whether there might also be a differential risk of CVD later in life.

All analyses were conducted using Stata version 16 (StataCorp LLC). The significance threshold was 5% with 2-sided unpaired testing.

## Results

**Figure 1.**
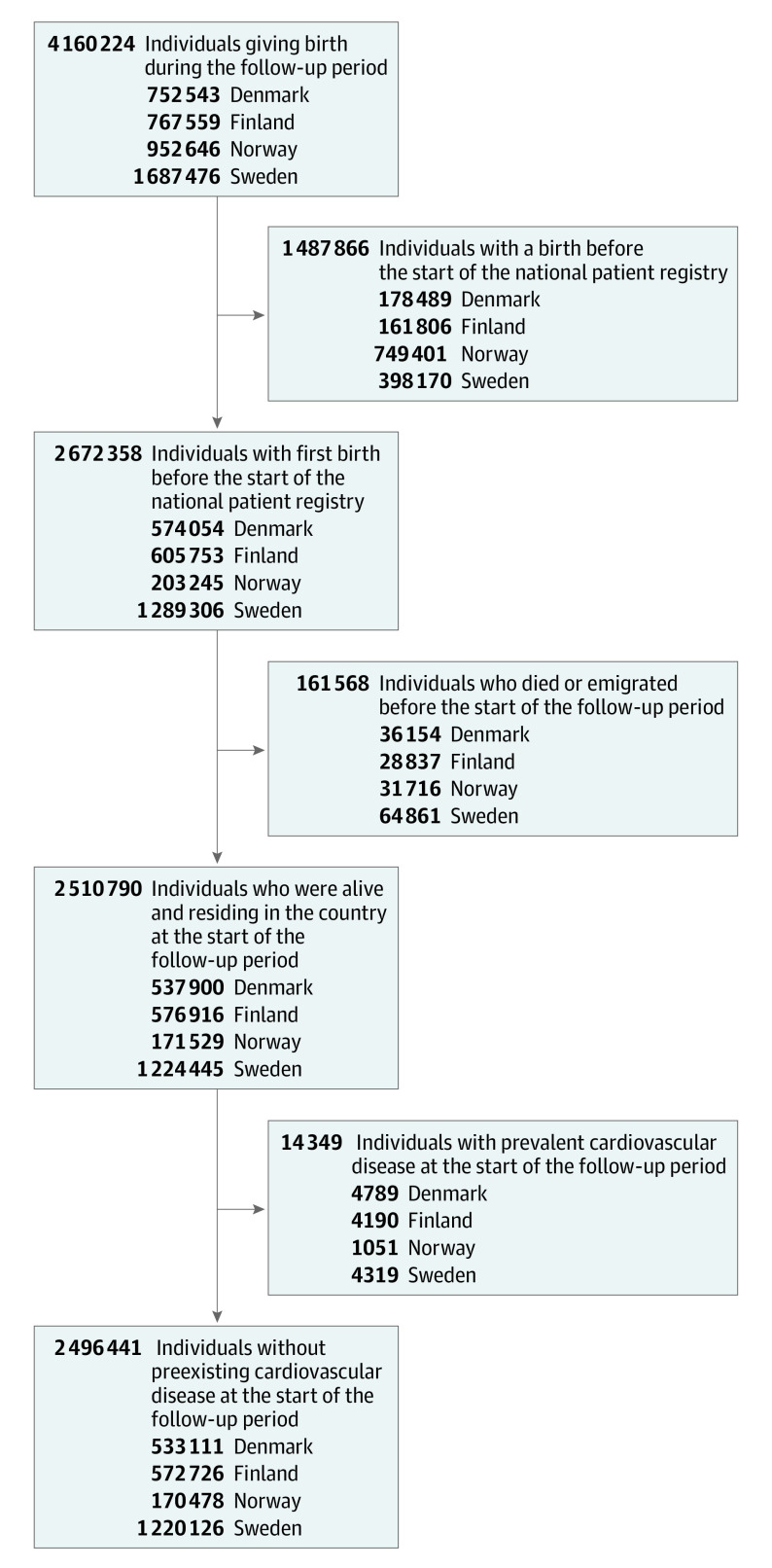
Illustration of Study Population

We excluded women who had given birth before the national patient registries started, those who had died or emigrated before the start of follow-up, and women with preexisting CVD (Figure 1). The study population therefore consisted of 2 496 441 nulliparous women at the start of follow-up without preexisting CVD, of whom 97 474 (4%) gave birth after ART by the end of follow-up. Individuals who gave birth after ART were older, had a lower parity, and were less likely to use tobacco compared with those who gave birth without ART (Table). The median follow-up time for all individuals was 11 (IQR, 5-18) years. Background characteristics were largely similar across the 4 countries (eTable 3 in Supplement 1).

**Table.** Background Characteristics According to Use of ART

Background characteristic	No. (%)	
	No use of ART (n = 2 398 967)	Use of ART (n = 97 474)
Age at start of follow-up, mean (SD), y	29.1 (4.9)	33.8 (4.7)
Parity at end of follow-up		
1	655 872 (27.3)	43 886 (45.0)
2	1 210 734 (50.5)	41 983 (43.1)
3	409 612 (17.1)	9561 (9.8)
≥4	122 749 (5.1)	2044 (2.1)
Prepregnancy BMI		
Underweight (<18.5)	60 671 (2.5)	1755 (1.8)
Normal weight (18.5-24.9)	993 625 (41.4)	42 607 (43.7)
Overweight (25.0-29.9)	312 050 (13.0)	15 330 (15.7)
Obesity (≥30.0)	138 931 (5.8)	6119 (6.3)
Missing	893 690 (37.3)	31 663 (32.5)
Tobacco use at start of follow-up		
No	1 939 475 (80.8)	85 845 (88.1)
Yes	331 914 (13.8)	6168 (6.3)
Missing	125 578 (5.2)	5461 (5.6)
Diabetes at start of follow-up		
No	2 385 563 (99.4)	96 758 (99.3)
Yes	13 404 (0.6)	716 (0.7)
Chronic hypertension at start of follow-up		
No	2 379 080 (99.2)	96 320 (98.8)
Yes	19.887 (0.8)	1154 (1.2)
PCOS		
No	2 384 890 (99.4)	93 606 (96.0)
Yes	14 077 (0.6)	3868 (4.0)
Highest obtained educational level		
Primary/secondary	1 129 385 (47.1)	38 073 (39.1)
Short tertiary/bachelor	652 067 (27.2)	29 857 (30.6)
Long tertiary/master or higher	321 367 (13.4)	18 254 (18.7)
Missing	296 148 (12.3)	11 290 (11.6)
Any preterm birth by end of follow-up		
No	2 168 179 (90.4)	80 156 (82.2)
Yes	230 788 (9.6)	17 318 (17.8)
Any small gestational age delivery by end of follow-up		
No	2 234 204 (93.1)	88 600 (90.9)
Yes	164 763 (6.9)	8.874 (9.1)
Any stillbirth by end of follow-up		
No	2 382 159 (99.3)	96 421 (98.9)
Yes	16 808 (0.7)	1053 (1.1)
Any history of pregnancy complicated by hypertensive disorders of pregnancy by end of follow-up		
No	2 207 323 (92.0)	87 435 (89.7)
Yes	191 644 (8.0)	10 039 (10.3)
Any multiple gestation by end of follow-up		
No	2 337 477 (97.4)	80 247 (82.3)
Yes	61 490 (2.6)	17 227 (17.7)

Abbreviations: ART, assisted reproductive technology; BMI, body mass index (calculated as weight in kilograms divided by height in meters squared); PCOS, polycystic ovary syndrome.

**Figure 2.**
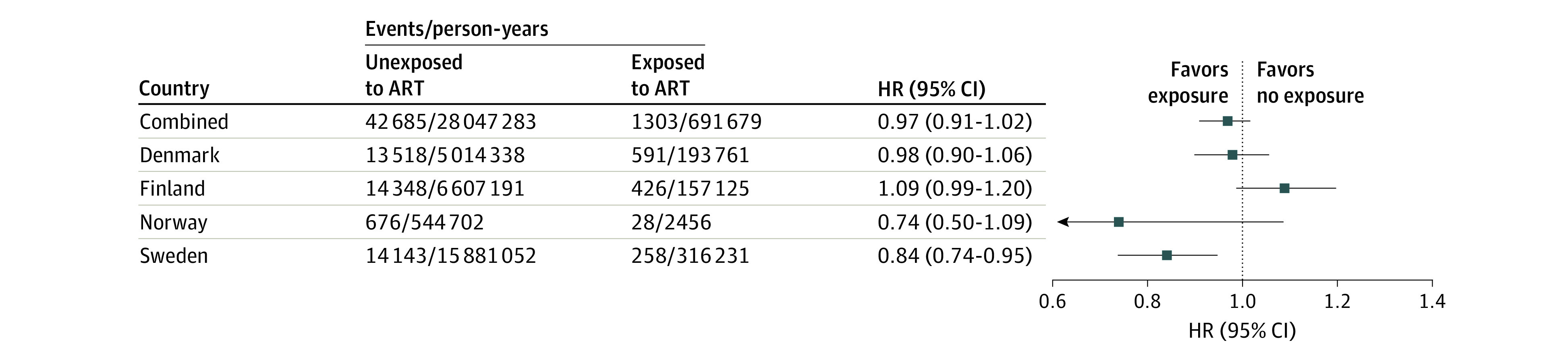
Risk of Any Cardiovascular Disease According to Conception by Assisted Reproductive Technologies (ARTs) Any cardiovascular diseases is defined as ischemic heart disease, cerebrovascular disease, cardiomyopathy, heart failure, pulmonary embolism, and deep vein thrombosis. Adjusted for age and parity, chronic hypertension, diabetes, polycystic ovary syndrome, and calendar year of start of follow-up, in addition to country in the combined model. HR indicates hazard ratio.

The rate of any CVD during follow-up was 153 per 100 000 person-years. We observed no significant difference in the risk of any CVD across the Nordic countries between individuals who delivered after using ART compared with those who delivered without use of ART (incidence rates, 188 vs 152 per 100 000 person-years), with an adjusted HR (AHR) of 0.97 (95% CI, 0.91-1.02) (Figure 2; eTable 4 in Supplement 1). There was, however, evidence of heterogeneity between countries (*I*^2^ = 76%; *P* = .01 for heterogeneity), with an AHR of 0.74 for Norway, 0.84 for Sweden, 0.98 for Denmark, and 1.09 for Finland (Figure 2; eTable 4 in Supplement 1). Adjustment for multiple gestation and pregnancy complications did not change our findings (eTable 4 in Supplement 1).

**Figure 3.**
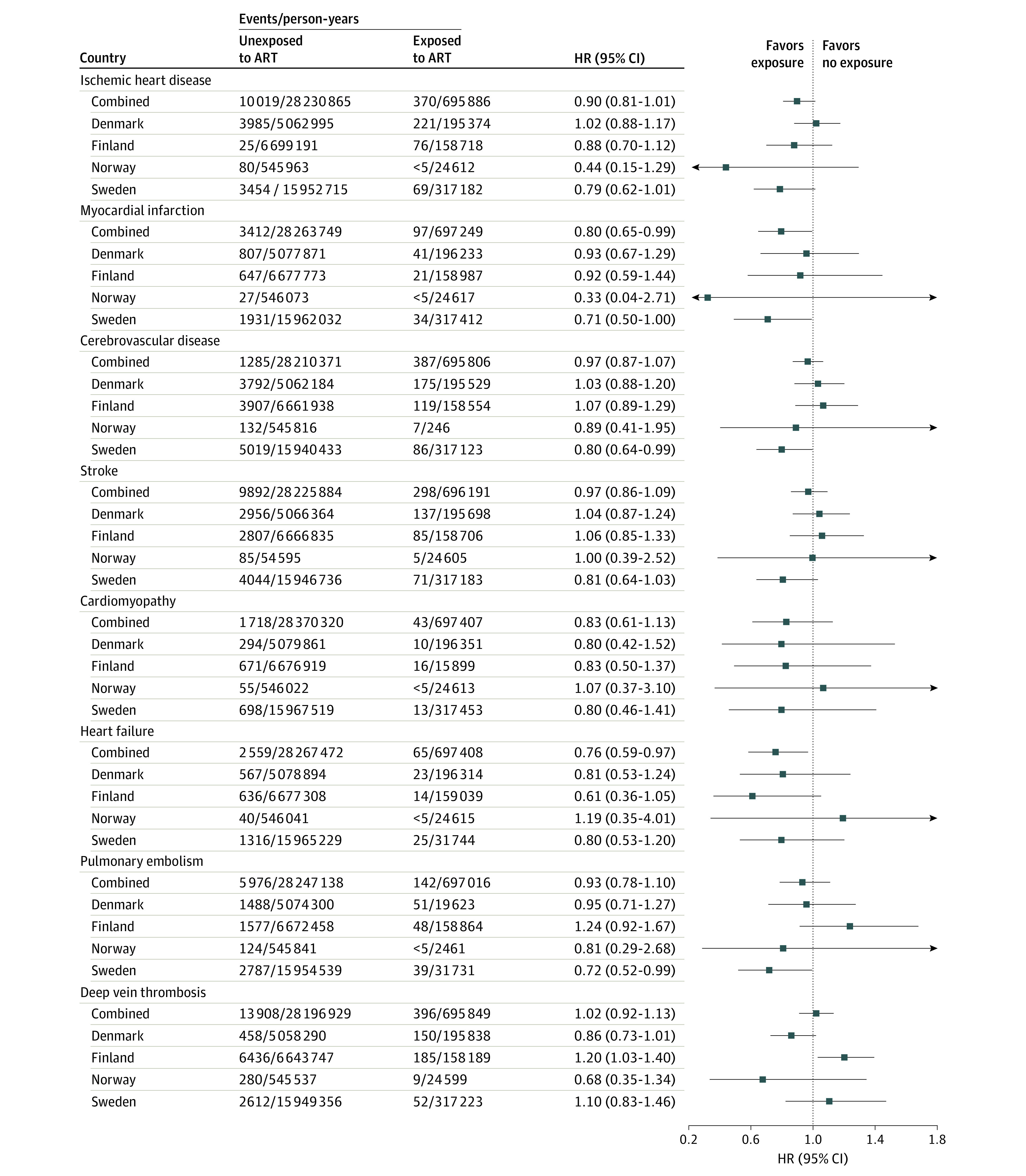
Risk of Subgroups of Cardiovascular Disease According to Conception by Assisted Reproductive Technologies (ARTs) Adjusted for age and parity, chronic hypertension, diabetes, polycystic ovary syndrome, and calendar year of start of follow-up, in addition to country in the combined model. HR indicates hazard ratio.

When examining subgroups of CVD, we observed no significant difference between women who delivered with and without using ART in the risk of ischemic heart disease, cerebrovascular disease, stroke, cardiomyopathy, heart failure, pulmonary embolism, or deep vein thrombosis (Figure 3; eTable 5 in Supplement 1). However, there was a lower risk of myocardial infarction (incidence rates, 14 vs 12 per 100 000; combined HR, 0.80; 95% CI, 0.65-0.99) among individuals who had used ART (Figure 3; eTable 5 in Supplement 1). Adjustment for multiple gestation and pregnancy complications did not change our findings (eTable 5 in Supplement 1). There was between-country heterogeneity in the association with deep vein thrombosis only (*I*^2^ = 71%; *P* = .02 for heterogeneity). When we further explored the source of heterogeneity in the estimates between countries, by excluding each country in turn, we found that Finland was the main source of this (eTable 6 in Supplement 1).

Information on tobacco use and body mass index was available for 50% of individuals across the 4 countries. Additional adjustment for these 2 covariates did not change the estimated association between use of ART and any CVD (eTable 7 in Supplement 1); the HR adjusted for the other covariates in this subsample was 1.00 (95% CI, 0.92-1.09) compared with 1.03 (95% CI, 0.95-1.12) after further adjustment for tobacco use and body mass index. The same was the case for the subgroups of CVD. Information on educational level was available for 93% of the study population from Denmark, Finland, and Sweden. Additional adjustment for educational level (eTable 8 in Supplement 1) also did not alter the results.

Excluding pulmonary embolism and deep vein thrombosis from the definition of any CVD yielded similar results (eTable 9 in Supplement 1). We observed no increased risk of CVD among women who had delivered after using ART when including women who had 1 or more deliveries before the start of follow-up in the analysis (eTable 10 in Supplement 1). When we stratified by year of start of follow-up, there was no increased risk of CVD among individuals who had delivered using ART in any of the groups (eTable 11 in Supplement 1).

**Figure 4.**
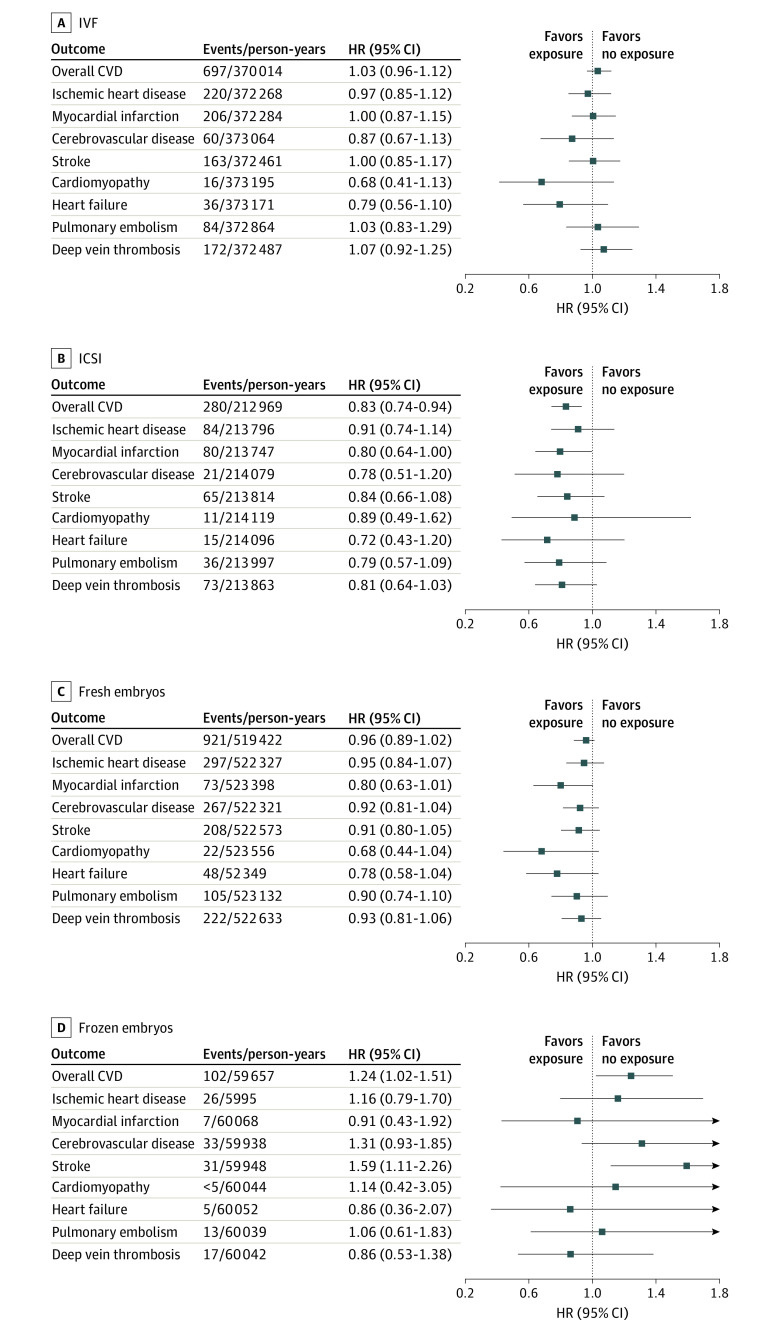
Risk of Cardiovascular Disease According to Methods of Conception by Assisted Reproductive Technologies (ARTs) Risk shown for in vitro fertilization (IVF) (A), intracytoplasmic sperm injection (ICSI) (B), fresh embryos (C), and frozen embryos (D). Adjusted for age and parity, chronic hypertension, diabetes, polycystic ovary syndrome, and calendar year of start of follow-up, in addition to country in the combined model. CVD indicates cardiovascular disease; HR, hazard ratio.

When estimating the risk of CVD according to ART fertilization method, we observed a lower risk for any CVD among individuals who had used ICSI, as compared with women who had not used ART (Figure 4). A lower risk among individuals who had used ICSI was also observed for the other outcomes, although the CIs were wide and included the null value (Figure 4). We observed weak evidence that frozen, but not fresh, embryo transfers were associated with an increased risk of stroke (HR, 1.59 [95% CI, 1.11-2.26] for frozen embryo transfer and 0.91 [95% CI, 0.80-1.05] for fresh embryo transfer) with nonoverlapping CIs (Figure 4). The association between frozen embryo transfer and risk of stroke persisted after further adjustment for pregnancy complications (AHR, 1.58 [95% CI, 1.10-1.04]).

## Discussion

In this Nordic registry-based study, women who had delivered after ART had no increased risk of CVD. We observed differences according to ART methods, with an increased risk of stroke among individuals who had used frozen but not fresh embryo transfer, and a decreased risk of most of the CVD subtypes among women who had used ICSI but not among those who had used IVF without ICSI. The results for the different ART methods were imprecise, and larger studies with longer follow-up are needed. Potential explanations for heterogeneity in the estimates between countries include differences in our ability to capture CVD across the countries (particularly outpatient diagnoses) and variation in the predominant ART methods used (no information on ART method was available for Finland to compare with the other countries).

Our results for any ART contrast with the findings from the meta-analysis^9^ suggesting a higher risk of cerebrovascular disease among individuals who used ART. Notably, in line with what was observed in the meta-analysis, we noted an elevated risk of stroke among individuals who had used frozen embryo transfer. This might reflect an increased risk of hypertensive disorders of pregnancy,^14^ since preeclampsia is associated with future risk of stroke.^15^ A higher risk of hypertensive disorders in pregnancy after frozen embryo transfer cycles may partly reflect an absence of corpus luteum in programmed cycles,^16,17^ although only 15% to 30% of frozen embryo transfer cycles were programmed in the Nordic countries during the study period.^18,19^ Our findings were similar after additional adjustment for pregnancy complications. Other potential explanations include a role of embryo selection,^20^ in addition to epigenetic changes associated with freezing or thawing,^21,22,23^ which may influence trophoblast invasion and placentation.^24^ However, a higher risk of CVD among individuals who have used frozen embryo transfer can also reflect a greater severity of the couple’s fertility problems, as they have usually had an unsuccessful fresh embryo transfer attempt before proceeding to frozen embryo transfer. However, frozen embryo transfer requires that multiple eggs have been harvested and successfully fertilized, which might reflect a higher fertility among this subgroup.

We further observed a decreased risk of most CVD subtypes among women who had used ICSI. A priori, we had hypothesized that if we observed any increased risk only among those who used IVF without ICSI, this was more likely to reflect a role of confounding by underlying female fertility problems, as ICSI is most often use in relation to male fertility problems in the Nordic countries.^2^ If we had observed an increased risk of a similar magnitude for IVF with and without ICSI, this would have supported the hypothesis that there was something related to the ART procedure itself. We observed neither of these scenarios. We speculate that women who used IVF with ICSI did not have fertility problems themselves, as this reflects largely fertility problems among their partners, they might be reproductively healthier, which again might be reflected in their decreased risk of CVD.

### Strengths and Limitations

Strengths of our study include the population-based design, the large sample, inclusion of 4 Nordic countries, the evaluation of CVD subgroups, and the comparison of ART methods. The universal health care service in the Nordic countries also ensures a comprehensive capture of both the use of ART and CVD. Our study also has limitations. Individuals who gave birth after ART are still young (median age at the end of follow-up, 41; IQR, 35-47 years), and we could therefore only study CVD risk up to middle age. We relied on the patient and cause of death registries for capturing CVD events, and validation studies indicate high accuracy in the registrations of relevant *ICD* codes when compared with medical records.^25,26,27^ We only captured deliveries after ART, and could not examine the risk of CVD among women who had only undergone ART and remained nulliparous. Notably, among those attempting ART, the proportion of couples who eventually succeed is approximately 2 of 3.^28,29^ We only had educational level as a measure of socioeconomic status, and no information on other measures, such as income, employment, or other markers. This could have resulted in residual confounding by socioeconomic status. We had no information on number and time for unsuccessful ART, and exposure status and time may have been misclassified, likely biasing the results toward the null. We only had information on tobacco use and body mass index for a subgroup of participants. However, additional adjustment for the subgroup with this information available did not change the results. We did not have a sufficient number of events to evaluate CVD risk according to the number of ART deliveries to explore a potential dose-response relationship. Unfortunately, we could not reliably separate gestational diabetes from prepregnancy diabetes due to different registration practices between the study countries and over time. We also cannot exclude a role of multiple comparisons, as we evaluated several CVD subtypes. Future studies with longer follow-up time and more CVD events should therefore evaluate whether the risk of CVD might vary according to the number of deliveries after ART.

## Conclusions

The findings of this cohort study suggest that women who gave birth after ART were not at increased risk of CVD over a median follow-up of 11 years compared with those who conceived without ART. These findings may be reassuring to the increasing number of individuals who require assistance from ART to conceive. Future studies with longer follow-up may be useful to reexamine differences in the risk of CVD according to ART methods.

## Notes

Supplement 1. **eTable 1.** Terms Related to Assisted Reproductive Technologies **eTable 2.** *International Classification of Diseases* Codes Used to Capture Cardiovascular Disease **eTable 3.** Background Characteristics According to Country **eTable 4.** Risk of Any Cardiovascular Disease According to Conception by Assisted Reproductive Technologies **eTable 5.** Risk of Subgroups of Cardiovascular Disease According to Conception by Assisted Reproductive Technologies **eTable 6.** Exploring Sources of Heterogeneity in the Risk of Cardiovascular Disease According to Conception by Assisted Reproductive Technologies by Excluding One Country in Turn **eTable 7.** Sensitivity Analysis of the Risk of Cardiovascular Disease According to Conception by Assisted Reproductive Technologies Adjusting for Tobacco Use and Body Mass Index **eTable 8.** Sensitivity Analysis of the Risk of Cardiovascular Disease According to Conception by Assisted Reproductive Technologies Adjusting for Education **eTable 9.** Risk of Any Cardiovascular Disease According to Conception by Assisted Reproductive Technologies Excluding Pulmonary Embolism and Deep Vein Thrombosis From the Outcome Definition **eTable 10.** Risk of Subgroups of Cardiovascular Disease According to Conception by Assisted Reproductive Technologies Also Including Parous Women at the Start of Follow-up **eTable 11.** Stratified Analysis of the Risk of Cardiovascular Disease According to Conception by Assisted Reproductive Technologies by Year of Start of Follow-up

Supplement 2.
Data Sharing Statement
