# Supplementary material for: Maternal Risk of Cardiovascular Disease After Use of Assisted Reproductive Technologies
Source: JAMA Cardiol. 2023 Aug 9;8(9):837–45. doi: 10.1001/jamacardio.2023.2324 (PMC10413220; doi:10.1001/jamacardio.2023.2324)
Supplement: Supplement 1. — eTable 1. Terms Related to Assisted Reproductive Technologies eTable 2. International Classification of Diseases Codes Used to Capture Cardiovascular Disease eTable 3. Background Characteristics According to Country eTable 4. Risk of Any Cardiovascular Disease According to Conception by Assisted Reproductive Technologies eTable 5. Risk of Subgroups of Cardiovascular Disease According to Conception by Assisted Reproductive Technologies eTable 6. Exploring Sources of Heterogeneity in the Risk of Cardiovascular Disease According to Conception by Assisted Reproductive Technologies by Excluding One Country in Turn eTable 7. Sensitivity Analysis of the Risk of Cardiovascular Disease According to Conception by Assisted Reproductive Technologies Adjusting for Tobacco Use and Body Mass Index eTable 8. Sensitivity Analysis of the Risk of Cardiovascular Disease According to Conception by Assisted Reproductive Technologies Adjusting for Education eTable 9. Risk of Any Cardiovascular Disease According to Conception by Assisted Reproductive Technologies Excluding Pulmonary Embolism and Deep Vein Thrombosis From the Outcome Definition eTable 10. Risk of Subgroups of Cardiovascular Disease According to Conception by Assisted Reproductive Technologies Also Including Parous Women at the Start of Follow-up eTable 11. Stratified Analysis of the Risk of Cardiovascular Disease According to Conception by Assisted Reproductive Technologies by Year of Start of Follow-up [file jamacardiol-e232324-s001.pdf]

Supplementary Online Content

Magnus MC, Fraser A, Håberg SE, et al. Maternal risk of cardiovascular disease after use of assisted reproductive technologies. *JAMA Cardiol*. Published online August 9, 2023. doi:10.1001/jamacardio.2023.2324

eTable 1. Terms Related to Assisted Reproductive Technologies

eTable 2. *International Classification of Diseases* Codes Used to Capture Cardiovascular Disease

eTable 3. Background Characteristics According to Country

eTable 4. Risk of Any Cardiovascular Disease According to Conception by Assisted Reproductive Technologies

eTable 5. Risk of Subgroups of Cardiovascular Disease According to Conception by Assisted Reproductive Technologies

eTable 6. Exploring Sources of Heterogeneity in the Risk of Cardiovascular Disease According to Conception by Assisted Reproductive Technologies by Excluding One Country in Turn

eTable 7. Sensitivity Analysis of the Risk of Cardiovascular Disease According to Conception by Assisted Reproductive Technologies Adjusting for Tobacco Use and Body Mass Index

eTable 8. Sensitivity Analysis of the Risk of Cardiovascular Disease According to Conception by Assisted Reproductive Technologies Adjusting for Education

eTable 9. Risk of Any Cardiovascular Disease According to Conception by Assisted Reproductive Technologies Excluding Pulmonary Embolism and Deep Vein Thrombosis From the Outcome Definition

eTable 10. Risk of Subgroups of Cardiovascular Disease According to Conception by Assisted Reproductive Technologies Also Including Parous Women at the Start of Follow-up

eTable 11. Stratified Analysis of the Risk of Cardiovascular Disease According to Conception by Assisted Reproductive Technologies by Year of Start of Follow-up

This supplementary material has been provided by the authors to give readers additional information about their work.

© 2023 American Medical Association. All rights reserved.

eTable 1 Terms related to assisted reproductive technologies

Term	Definition [a]
Assisted reproductive technologies (ART)	All interventions that include the in vitro handling of both human oocytes and sperm or of embryos for the purpose of reproduction. This includes, but is not limited to, in vitro fertilization (IVF) and embryo transfer, intracytoplasmic sperm injection (ICSI), embryo biopsy, preimplantation genetic testing, assisted hatching, gamete intrafallopian transfer, zygote intrafallopian transfer, gamete and embryo cryopreservation, semen, oocyte and embryo donation, and gestational carrier cycles.
Frozen embryo transfer (FET) cycle	An ART procedure in which cycle monitoring is carried out with the intention of transferring to a woman, frozen/thawed or vitrified/warmed embryo(s)/blastocyst(s). Note: A FET cycle is initiated when specific medication is provided or cycle monitoring is started in the female recipient with the intention to transfer an embryo.
In vitro fertilization (IVF)	A sequence of procedures that involves extracorporeal fertilization of gametes. It includes conventional in vitro insemination and ICSI.
Intracytoplasmic sperm injection (ICSI)	A procedure in which a single spermatozoon is injected into the oocyte cytoplasm.

[a] The definitions are taken from the International Glossary on Infertility and Fertility Care, published in Human Reproduction by Zegers-Hochschold et. al in 2017.

© 2023 American Medical Association. All rights reserved.

eTable 2 International classification of diseases codes used to capture cardiovascular disease

	Version 10	Version 9	Version 8
Ischemic heart disease	I20-I25	410-414	410-414
Cerebrovascular disease	I60-I69	430-438	430-438
Myocardial infarction	I21, I22	410	410
Stroke	I60, I61, I63, I64	430-434, 436	430-434, 436
Cardiomyopathy	I42, I43	425	425
Heart failure	I50	428	428
Pulmonary embolism	I26	415B	450
Deep vein thrombosis	I80, I82	451, 453	451, 453

© 2023 American Medical Association. All rights reserved.

eTable 3 Background characteristics according to country

Background characteristics	Denmark (n= 533,111 women)	Finland (n= 572,726 women)	Norway (n= 170,478 women)	Sweden (n= 1,220,126 women)
**Age at start of follow-up, mean(SD)**	29.6 (4.7)	28.9 (5.1)	30.0 (5.1)	29.1 (5.0)
**Number of years of follow-up median (IQR)**	10 (5, 15)	12 (6, 19)	3 (2, 5)	13 (6, 21)
**Parity at end of follow-up, N(%)**				
1	152,497 (28.6)	162,454 (28.4)	77,661 (45.6)	307,146 (25.2)
2	274,172 (51.4)	261,617 (45.7)	78,831 (46.2)	638,097 (52.3)
3	85,169 (16.0)	105,760 (18.5)	12,664 (7.4)	215,580 (17.7)
4 or higher	21,273 (4.0)	42,895 (7.5)	1,322 (0.8)	59,303 (4.9)
**Used ART during follow-up, N(%)**				
No	505,670 (94.9)	552,994 (96.6)	162,449 (95.3)	1,177,854 (96.5)
Yes	27,441 (5.2)	19,732 (3.5)	8,029 (4.7)	42,272 (3.5)
**Pre-pregnancy body-mass index, N(%)**				
Underweight (<18.5 kg/m^2)	11,527 (2.2)	9,904 (1.7)	4,526 (2.7)	36,469 (3.0)
Normal weight (18.5-24.9 kg/m^2)	160,356 (30.1)	150,355 (26.3)	58,159 (34.1)	667,362 (54.7)
Overweight (25-29.9 kg/m^2)	49,759 (9.3)	47,581 (8.3)	19,014 (11.2)	211,026 (17.3)
Obesity (≥30 kg/m^2)	27,308 (5.1)	24,589 (4.3)	10,102 (5.9)	83,051 (6.8)
Missing	284,161 (53.3)	340,297 (59.4)	78,677 (46.2)	222,218 (18.2)
**Tobacco use at start of follow-up, N(%)**				
No	412,150 (77.3)	468,753 (81.9)	128,506 (75.4)	1,015,911 (83.3)
Yes	77,820 (14.6)	94,413 (16.5)	16,508 (9.7)	149,341 (12.2)
Missing	43,141 (8.1)	9,560 (1.7)	25,464 (14.9)	54,874 (4.5)
**Diabetes at start of follow-up, N(%)**				
No	530,215 (99.5)	569,205 (99.4)	169,074 (99.2)	1,213,837 (99.5)
Yes	2,896 (0.5)	3,521 (0.6)	1,404 (0.8)	6,299 (0.5)
**Chronic hypertension at start of follow-up, N(%)**				
No	528,637 (99.2)	563,580 (98.4)	169,634 (99.5)	1,213,549 (99.5)
Yes	4,474 (0.8)	9,146 (1.6)	844 (0.5)	6,577 (0.5)
**PCOS, N(%)**				
No	528,787 (99.2)	570,056 (99.5)	167,890 (98.5)	1,211,763 (99.3)
Yes	4,324 (0.8)	2,670 (0.5)	2,588 (1.5)	8,363 (0.7)
**Highest obtained educational level, N(%)**				
Primary/secondary	308,418 (57.9)	224,046 (39.1)	NA	634,994 (52.0)

© 2023 American Medical Association. All rights reserved.

Short tertiary/bachelor	143,382 (26.9)	191,249 (33.4)	NA	347,294 (28.5)
Long tertiary/master or higher	69,736 (13.1)	104,006 (18.2)	NA	165,879 (13.6)
Missing	11,575 (2.2)	53,425 (9.3)	NA	71,960 (5.9)
**Any preterm birth by end of follow-up, N(%)**				
No	477,343 (89.5)	517,835 (90.4)	155,504 (91.2)	1,097,653 (90.0)
Yes	55,768 (10.5)	54,891 (9.6)	14,974 (8.8)	122,473 (10.0)
**Any small-gestational-age delivery by end of follow-up, N(%)**				
No	491,998 (92.3)	533,149 (93.1)	159,841 (93.8)	1,137,816 (93.3)
Yes	41,114 (7.7)	39,577 (6.9)	10,637 (6.2)	82,310 (6.8)
**Any stillbirth by end of follow-up, N(%)**				
No	528,876 (99.2)	568,863 (99.3)	169,049 (99.2)	1,211,792 (99.3)
Yes	4,235 (0.8)	3,863 (0.7)	1,429 (0.8)	8,334 (0.7)
**Any history of pregnancy complicated by hypertensive disorders of pregnancy by end of follow-up, N(%)**				
No	495,122 (92.9)	508,727 (88.8)	157,042 (92.1)	1,133,867 (92.9)
Yes	37,989 (7.1)	63,999 (11.2)	13,436 (7.9)	86,259 (7.1)
**Any multiple birth by end of follow-up, N(%)**				
No	511,377 (95.9)	555,001 (96.9)	165,891 (97.3)	1,185,455 (97.2)
Yes	21,734 (4.1)	17,725 (3.1)	4,587 (2.7)	34,671 (2.8)

© 2023 American Medical Association. All rights reserved.

eTable 4 Risk of any cardiovascular disease according to conception by assisted reproductive technologies

Country	Exposure	N follow-up time in years	N events	Age and calendar year adjusted HR (95% CI)	Adjusted [a] HR (95% CI)	Adjusted [b] HR (95% CI)
Combined	Non-ART	28,047,283	42,685	Ref	Ref	Ref
	ART	691,679	1,303	1.04 (0.99-1.10)	0.97 (0.91-1.02)	0.96 (0.91-1.02)
Denmark	Non-ART	5,014,338	13,518	Ref	Ref	Ref
	ART	193,761	591	1.01 (0.93-1.10)	0.98 (0.90-1.06)	0.97 (0.89-1.06)
Finland	Non-ART	6,607,191	14,348	Ref	Ref	Ref
	ART	157,125	426	1.10 (1.00-1.21)	1.09 (0.99-1.20)	1.07 (0.96-1.18)
Norway	Non-ART	544,702	676	Ref	Ref	Ref
	ART	24,560	28	0.76 (0.52-1.11)	0.74 (0.50-1.09)	0.73 (0.49-1.07)
Sweden	Non-ART	15,881,052	14,143	Ref	Ref	Ref
	ART	316,231	258	0.88 (0.78-1.00)	0.84 (0.74-0.95)	0.83 (0.73-0.95)

[a] Adjusted for age, parity, polycystic ovarian syndrome, diabetes, chronic hypertension, year of start of follow-up, in addition to country (in the combined model).
[b] Adjusted for age, parity, polycystic ovarian syndrome, diabetes, chronic hypertension, year of start of follow-up, country (in the combined model), multiple gestation, preterm birth, small-for-gestational-age birth, stillbirth and hypertensive disorders of pregnancy.

© 2023 American Medical Association. All rights reserved.

eTable 5 Risk of subgroups of cardiovascular disease according to conception by assisted reproductive technologies

Outcome	Country	Exposure	N follow-up time in years	N events	Age and calendar year adjusted HR (95% CI)	Adjusted [a] HR (95% CI)	Adjusted [b] HR (95% CI)
Ischemic heart disease	Combined	Non-ART	28,230,865	10,019	Ref	Ref	Ref
		ART	695,886	370	1.03 (0.93-1.15)	0.90 (0.81-1.01)	0.91 (0.81-1.02)
	Denmark	Non-ART	5,062,995	3,985	Ref	Ref	Ref
		ART	195,374	221	1.08 (0.94-1.24)	1.02 (0.88-1.17)	1.05 (0.90-1.21)
	Finland	Non-ART	6,699,191	2,500	Ref	Ref	Ref
		ART	158,718	76	0.89 (0.71-1.13)	0.88 (0.70-1.12)	0.84 (0.66-1.06)
	Norway	Non-ART	545,963	80	Ref	Ref	Ref
		ART	24,612	<5	0.57 (0.21-1.59)	0.44 (0.15-1.29)	0.43 (0.15-1.26)
	Sweden	Non-ART	15,952,715	3,454	Ref	Ref	Ref
		ART	317,182	69	0.87 (0.68-1.11)	0.79 (0.62-1.01)	0.78 (0.61-1.01)
Myocardial Infarction	Combined	Non-ART	28,263,749	3,412	Ref	Ref	Ref
		ART	697,249	97	0.91 (0.74-1.12)	0.80 (0.65-0.99)	0.82 (0.66-1.02)
	Denmark	Non-ART	5,077,871	807	Ref	Ref	Ref
		ART	196,233	41	1.03 (0.74-1.42)	0.93 (0.67-1.29)	0.98 (0.70-1.38)
	Finland	Non-ART	6,677,773	647	Ref	Ref	Ref
		ART	158,987	21	0.95 (0.61-1.49)	0.92 (0.59-1.44)	0.94 (0.60-1.49)
	Norway	Non-ART	546,073	27	Ref	Ref	Ref
		ART	24,617	<5	0.40 (0.05-2.99)	0.33 (0.04-2.71)	0.32 (0.04-2.57)
	Sweden	Non-ART	15,962,032	1,931	Ref	Ref	Ref
		ART	317,412	34	0.78 (0.55-1.10)	0.71 (0.50-1.00)	0.69 (0.48-0.99)
Cerebrovascular disease	Combined	Non-ART	28,210,371	12,850	Ref	Ref	Ref
		ART	695,806	387	1.05 (0.94-1.16)	0.97 (0.87-1.07)	0.97 (0.87-1.08)
	Denmark	Non-ART	5,062,184	3,792	Ref	Ref	Ref
		ART	195,529	175	1.09 (0.93-1.27)	1.03 (0.88-1.20)	1.03 (0.87-1.21)
	Finland	Non-ART	6,661,938	3,907	Ref	Ref	Ref
		ART	158,554	119	1.09 (0.90-1.32)	1.07 (0.89-1.29)	1.08 (0.89-1.31)
	Norway	Non-ART	545,816	132	Ref	Ref	Ref
		ART	24,600	7	0.88 (0.41-1.92)	0.89 (0.41-1.95)	0.87 (0.39-1.91)
	Sweden	Non-ART	15,940,433	5,019	Ref	Ref	Ref
		ART	317,123	86	0.82 (0.66-1.02)	0.80 (0.64-0.99)	0.77 (0.61-0.96)
Stroke	Combined	Non-ART	28,225,884	9,892	Ref	Ref	Ref

© 2023 American Medical Association. All rights reserved.

		ART	696,191	298	1.05 (0.94-1.18)	0.97 (0.86-1.09)	0.97 (0.86-1.10)
	Denmark	Non-ART	5,066,364	2,956	Ref	Ref	Ref
		ART	195,698	137	1.11 (0.93-1.33)	1.04 (0.87-1.24)	1.06 (0.88-1.27)
	Finland	Non-ART	6,666,835	2,807	Ref	Ref	Ref
		ART	158,706	85	1.08 (0.86-1.35)	1.06 (0.85-1.33)	1.09 (0.87-1.36)
	Norway	Non-ART	545,950	85	Ref	Ref	Ref
		ART	24,605	5	0.95 (0.38-2.39)	1.00 (0.39-2.52)	0.99 (0.39-2.52)
	Sweden	Non-ART	15,946,736	4,044	Ref	Ref	Ref
		ART	317,183	71	0.83 (0.66-1.06)	0.81 (0.64-1.03)	0.77 (0.60-0.87)
Cardiomyopathy	Combined	Non-ART	28,370,320	1,718	Ref	Ref	Ref
		ART	697,407	43	0.84 (0.62-1.15)	0.83 (0.61-1.13)	0.81 (0.59-1.12)
	Denmark	Non-ART	5,079,861	294	Ref	Ref	Ref
		ART	196,351	10	0.77 (0.41-1.47)	0.80 (0.42-1.52)	0.72 (0.36-1.45)
	Finland	Non-ART	6,676,919	671	Ref	Ref	Ref
		ART	158,990	16	0.84 (0.50-1.38)	0.83 (0.50-1.37)	0.75 (0.45-1.27)
	Norway	Non-ART	546,022	55	Ref	Ref	Ref
		ART	24,613	<5	1.15 (0.40-3.26)	1.07 (0.37-3.10)	1.04 (0.35-3.05)
	Sweden	Non-ART	15,967,519	698	Ref	Ref	Ref
		ART	317,453	13	0.84 (0.48-1.47)	0.80 (0.46-1.41)	0.88 (0.50-1.56)
Heart failure	Combined	Non-ART	28,267,472	2,559	Ref	Ref	Ref
		ART	697,408	65	0.83 (0.64-1.06)	0.76 (0.59-0.97)	0.78 (0.60-1.01)
	Denmark	Non-ART	5,078,894	567	Ref	Ref	Ref
		ART	196,314	23	0.86 (0.56-1.32)	0.81 (0.53-1.24)	0.89 (0.57-1.39)
	Finland	Non-ART	6,677,308	636	Ref	Ref	Ref
		ART	159,039	14	0.64 (0.37-1.10)	0.61 (0.36-1.05)	0.58 (0.34-1.01)
	Norway	Non-ART	546,041	40	Ref	Ref	Ref
		ART	24,615	<5	1.24 (0.37-4.15)	1.19 (0.35-4.01)	1.03 (0.30-3.56)
	Sweden	Non-ART	15,965,229	1,316	Ref	Ref	Ref
		ART	317,440	25	0.86 (0.57-1.28)	0.80 (0.53-1.20)	0.83 (0.55-1.24)
Pulmonary embolism	Combined	Non-ART	28,247,138	5,976	Ref	Ref	Ref
		ART	697,016	142	0.98 (0.83-1.16)	0.93 (0.78-1.10)	0.93 (0.78-1.11)
	Denmark	Non-ART	5,074,300	1,488	Ref	Ref	Ref
		ART	196,230	51	0.98 (0.73-1.30)	0.95 (0.71-1.27)	0.93 (0.69-1.25)
	Finland	Non-ART	6,672,458	1,577	Ref	Ref	Ref
		ART	158,864	48	1.25 (0.93-1.68)	1.24 (0.92-1.67)	1.25 (0.92-1.69)
	Norway	Non-ART	545,841	124	Ref	Ref	Ref
		ART	24,610	<5	0.87 (0.31-2.40)	0.81 (0.29-2.68)	0.85 (0.30-2.38)

© 2023 American Medical Association. All rights reserved.

	Sweden	Non-ART	15,954,539	2,787	Ref	Ref	Ref
		ART	317,310	39	0.75 (0.54-1.04)	0.72 (0.52-0.99)	0.73 (0.53-1.01)
Deep vein thrombosis	Combined	Non-ART	28,196,929	13,908	Ref	Ref	Ref
		ART	695,849	396	1.07 (0.97-1.19)	1.02 (0.92-1.13)	0.99 (0.89-1.10)
	Denmark	Non-ART	5,058,290	4,580	Ref	Ref	Ref
		ART	195,838	150	0.86 (0.73-1.02)	0.86 (0.73-1.01)	0.82 (0.69-0.98)
	Finland	Non-ART	6,643,747	6,436	Ref	Ref	Ref
		ART	158,189	185	1.20 (1.03-1.39)	1.20 (1.03-1.40)	1.16 (1.00-1.35)
	Norway	Non-ART	545,537	280	Ref	Ref	Ref
		ART	24,599	9	0.66 (0.33-1.29)	0.68 (0.35-1.34)	0.67 (0.34-1.32)
	Sweden	Non-ART	15,949,356	2,612	Ref	Ref	Ref
		ART	317,223	52	1.18 (0.89-1.56)	1.10 (0.83-1.46)	1.12 (0.84-1.50)

[a] Adjusted for age parity, polycystic ovarian syndrome, diabetes, chronic hypertension, year of start of follow-up, in addition to country (in the combined model).
[b] Adjusted for age parity, polycystic ovarian syndrome, diabetes, chronic hypertension, year of start of follow-up, country (in the combined model), multiple gestation, preterm birth, small-for-gestational-age birth, stillbirth and hypertensive disorders of pregnancy.

© 2023 American Medical Association. All rights reserved.

eTable 6 Exploring sources of heterogeneity in the risk of cardiovascular disease according to conception by assisted reproductive technologies by excluding one country in turn

Outcome	Including all four countries<br>I^2, p-value	Excluding Denmark<br>I^2, p-value	Excluding Finland<br>I^2, p-value	Excluding Norway<br>I^2, p-value	Excluding Sweden<br>I^2, p-value
Any CVD	76%, 0.01	84%, 0.002	63%, 0.07	81%, 0.01	63%, 0.07
Ischemic Heart disease	45%, 0.15	0%, 0.42	61%, 0.08	43%, 0.17	38%, 0.20
Myocardial infarction	0%, 0.54	0%, 0.49	0%, 0.38	0%, 0.49	0%, 0.64
Cerebrovascular disease	35%, 0.20	50%, 0.14	42%, 0.18	56%, 0.10	0%, 0.88
Stroke	11%, 0.34	24%, 0.27	27%, 0.25	81%, 0.01	0%, 0.99
Cardiomyopathy	0%, 0.97	0%, 0.89	0%, 0.88	0%, 0.99	0%, 0.90
Heart failure	0%, 0.73	0%, 0.54	0%, 0.83	0%, 0.67	0%, 0.54
Pulmonary embolism	50%, 0.11	67%, 0.05	0%, 0.46	66%, 0.05	0%, 0.40
Deep vein thrombosis	71%, 0.02	27%, 0.25	31%, 0.23	77%, 0.01	80%, 0.01

© 2023 American Medical Association. All rights reserved.

eTable 7 Sensitivity analysis of the risk of cardiovascular disease according to conception by assisted reproductive technologies adjusting for tobacco use and body mass index

Outcome	Country	Exposure	N follow-up time in years	N events	Age and calendar year adjusted HR (95% CI)	Adjusted [a] HR (95% CI)	Adjusted [b] HR (95% CI)
Any CVD	Combined	Non-ART	19,377,836	23,086	Ref	Ref	Ref
		ART	433,339	612	1.04 (0.96-1.13)	1.00 (0.92-1.09)	1.03 (0.95-1.12)
	Denmark	Non-ART	2,470,912	6,057	Ref	Ref	Ref
		ART	85,820	232	1.05 (0.91-1.20)	1.04 (0.91-1.19)	1.06 (0.92-1.21)
	Finland	Non-ART	2,420,287	4,880	Ref	Ref	Ref
		ART	54,401	147	1.30 (1.09-1.52)	1.28 (1.08-1.52)	1.31 (1.10-1.55)
	Norway	Non-ART	309,851	390	Ref	Ref	Ref
		ART	14,300	15	0.69 (0.41-1.17)	0.66 (0.39-1.13)	0.68 (0.40-1.16)
	Sweden	Non-ART	14,176,787	11,759	Ref	Ref	Ref
		ART	278,817	218	0.92 (0.80-1.06)	0.88 (0.77-1.01)	0.90 (0.78-1.03)
Ischemic heart disease	Combined	Non-ART	19,476,745	4,697	Ref	Ref	Ref
		ART	435,142	131	0.92 (0.77-1.10)	0.87 (0.72-1.03)	0.91 (0.76-1.09)
	Denmark	Non-ART	2,490,944	1,427	Ref	Ref	Ref
		ART	86,358	68	1.10 (0.86-1.43)	1.08 (0.84-1.39)	1.11 (0.86-1.43)
	Finland	Non-ART	2,437,913	546	Ref	Ref	Ref
		ART	54,826	8	0.52 (0.26-1.05)	0.52 (0.26-1.06)	0.53 (0.26-1.08)
	Norway	Non-ART	310,529	45	Ref	Ref	Ref
		ART	14,323	<5	0.53 (0.13-2.23)	0.36 (0.08-1.68)	0.38 (0.08-1.75)
	Sweden	Non-ART	14,237,359	2,679	Ref	Ref	Ref
		ART	279,636	53	0.88 (0.67-1.17)	0.80 (0.61-1.06)	0.86 (0.65-1.13)
Cerebrovascular disease	Combined	Non-ART	19,461,441	7,036	Ref	Ref	Ref
		ART	434,920	186	1.06 (0.91-1.23)	1.01 (0.87-1.17)	1.04 (0.90-1.21)
	Denmark	Non-ART	2,489,043	1,622	Ref	Ref	Ref
		ART	86,305	68	1.18 (0.92-1.52)	1.14 (0.89-1.46)	1.16 (0.90-1.49)
	Finland	Non-ART	2,436,124	1,170	Ref	Ref	Ref
		ART	54,746	41	1.32 (0.96-1.83)	1.32 (0.95-1.82)	1.36 (0.98-1.88)
	Norway	Non-ART	310,464	70	Ref	Ref	Ref
		ART	14,321	<5	0.65 (0.20-2.11)	0.66 (0.20-2.17)	0.70 (0.22-2.29)

© 2023 American Medical Association. All rights reserved.

	Sweden	Non-ART	14,225,811	4,174	Ref	Ref	Ref
		ART	279,549	74	0.88 (0.70-1.11)	0.86 (0.68-1.08)	0.88 (0.70-1.12)
Myocardial Infarction	Combined	Non-ART	19,489,874	1,862	Ref	Ref	Ref
		ART	435,506	41	0.85 (0.62-1.16)	0.77 (0.56-1.06)	0.84 (0.61-1.16)
	Denmark	Non-ART	2,495,297	244	Ref	Ref	Ref
		ART	86,529	12	1.15 (0.63-2.09)	1.10 (0.60-2.00)	1.15 (0.63-2.10)
	Finland	Non-ART	2,439,736	92	Ref	Ref	Ref
		ART	54,855	<5	0.28 (0.04-2.06)	0.26 (0.04-1.94)	0.29 (0.04-2.11)
	Norway	Non-ART	310,588	13	Ref	Ref	Ref
		ART	14,323	<5	0.80 (0.10-6.42)	0.77 (0.09-6.80)	0.95 (0.11-8.16)
	Sweden	Non-ART	14,244,253	1,513	Ref	Ref	Ref
		ART	279,798	27	0.82 (0.56-1.21)	0.74 (0.50-1.09)	0.81 (0.55-1.19)
Stroke	Combined	Non-ART	19,469,849	5,383	Ref	Ref	Ref
		ART	435,069	143	1.07 (0.90-1.27)	1.03 (0.87-1.22)	1.06 (0.89-1.26)
	Denmark	Non-ART	2,490,858	1,212	Ref	Ref	Ref
		ART	86,367	52	1.25 (0.93-1.66)	1.20 (0.90-1.60)	1.22 (0.92-1.63)
	Finland	Non-ART	2,437,351	779	Ref	Ref	Ref
		ART	54,772	28	1.33 (0.90-1.98)	1.34 (0.90-1.98)	1.38 (0.93-2.05)
	Norway	Non-ART	310,526	47	Ref	Ref	Ref
		ART	14.321	<5	0.60 (1.14-2.54)	0.69 (0.16-2.91)	0.73 (0.17-3.06)
	Sweden	Non-ART	14,231,114	3,345	Ref	Ref	Ref
		ART	279,608	61	0.90 (0.69-1.16)	0.87 (0.67-1.13)	0.90 (0.69-1.16)
Cardiomyopathy	Combined	Non-ART	19,493,066	983	Ref	Ref	Ref
		ART	435,541	22	0.82 (0.53-1.26)	0.80 (0.52-1.24)	0.83 (0.54-1.28)
	Denmark	Non-ART	2,495,421	140	Ref	Ref	Ref
		ART	86,537	7	1.30 (0.59-2.83)	1.39 (0.63-3.04)	1.43 (0.65-3.13)
	Finland	Non-ART	2,439,255	206	Ref	Ref	Ref
		ART	54,849	<5	0.52 (0.16-1.65)	0.51 (0.16-1.63)	0.53 (0.17-1.69)
	Norway	Non-ART	310,557	34	Ref	Ref	Ref
		ART	14,322	2	0.78 (0.18-3.45)	0.75 (0.17-3.25)	0.74 (0.17-3.23)
	Sweden	Non-ART	14,247,833	603	Ref	Ref	Ref
		ART	279,832	10	0.75 (0.40-1.42)	0.72 (0.38-1.36)	0.74 (0.39-1.40)
Heart failure	Combined	Non-ART	19,491,409	1,425	Ref	Ref	Ref

© 2023 American Medical Association. All rights reserved.

		ART	435,535	37	1.04 (0.74-1.45)	0.98 (0.70-1.37)	1.03 (0.73-1.44)
	Denmark	Non-ART	2,495,292	196	Ref	Ref	Ref
		ART	86,541	9	1.34 (0.67-2.68)	1.36 (0.68-2.73)	1.41 (0.70-2.82)
	Finland	Non-ART	2,439,388	156	Ref	Ref	Ref
		ART	54,850	<5	0.76 (0.27-2.11)	0.71 (0.26-1.98)	0.75 (0.27-2.10)
	Norway	Non-ART	310,574	24	Ref	Ref	Ref
		ART	14,322	<5	1.99 (0.57-6.99)	1.83 (0.51-6.57)	1.81 (0.50-6.51)
	Sweden	Non-ART	14,246,155	1,049	Ref	Ref	Ref
		ART	279,823	21	0.95 (0.61-1.47)	0.89 (0.57-1.39)	0.93 (0.60-1.45)
Pulmonary embolism	Combined	Non-ART	19,477,727	3,832	Ref	Ref	Ref
		ART	435,316	75	0.89 (0.71-1.13)	0.86 (0.68-1.09)	0.87 (0.69-1.10)
	Denmark	Non-ART	2,492,722	805	Ref	Ref	Ref
		ART	86,518	21	0.80 (0.52-1.25)	0.80 (0.51-1.24)	0.80 (0.51-1.25)
	Finland	Non-ART	2,437,775	555	Ref	Ref	Ref
		ART	54,788	16	1.35 (0.81-2.26)	1.34 (0.80-2.24)	1.35 (0.81-2.26)
	Norway	Non-ART	310,449	78	Ref	Ref	Ref
		ART	14,320	<5	0.97 (0.30-3.18)	0.85 (0.26-2.85)	0.85 (0.26-2.85)
	Sweden	Non-ART	14,236,782	2,394	Ref	Ref	Ref
		ART	279,690	35	0.79 (0.56-1.11)	0.76 (0.54-1.07)	0.76 (0.54-1.08)
Deep vein thrombosis	Combined	Non-ART	19,457,409	7,292	Ref	Ref	Ref
		ART	434,885	198	1.14 (0.98-1.31)	1.11 (0.96-1.28)	1.13 (0.98-1.31)
	Denmark	Non-ART	2,486,304	2,294	Ref	Ref	Ref
		ART	86,356	67	0.86 (0.67-1.11)	0.88 (0.68-1.13)	0.90 (0.70-1.16)
	Finland	Non-ART	2,428,537	2,581	Ref	Ref	Ref
		ART	54,597	80	1.48 (1.17-1.85)	1.47 (1.17-1.85)	1.50 (1.19-1.88)
	Norway	Non-ART	310,295	160	Ref	Ref	Ref
		ART	14,318	<5	0.52 (0.19-1.44)	0.55 (0.20-1.50)	0.56 (0.20-1.53)
	Sweden	Non-ART	14,232,283	2,257	Ref	Ref	Ref
		ART	279,613	47	1.23 (0.92-1.66)	1.16 (0.86-1.57)	1.17 (0.86-1.57)

[a] Adjusted for age, parity, polycystic ovarian syndrome, chronic hypertension, diabetes, year of start of follow-up in addition to country (in the pooled model).
[b] Adjusted for age, parity, polycystic ovarian syndrome, chronic hypertension, diabetes, year of start of follow-up, country (in the combined model), in addition to tobacco use and body-mass index.

© 2023 American Medical Association. All rights reserved.

eTable 8 Sensitivity analysis of the risk of cardiovascular disease according to conception by assisted reproductive technologies adjusting for education

Outcome	Country	Exposure	N follow-up time in years	N events	Age and calendar year adjusted HR (95% CI)	Adjusted [a] HR (95% CI)	Adjusted [b] HR (95% CI)
Any CVD	Combined	Non-ART	26,287,103	39,862	Ref	Ref	Ref
		ART	646,897	1,238	1.05 (0.99-1.11)	0.97 (0.92-1.03)	0.96 (0.91-1.02)
	Denmark	Non-ART	4,969,445	13,389	Ref	Ref	Ref
		ART	192,714	588	1.01 (0.93-1.10)	0.98 (0.90-1.07)	0.96 (0.88-1.04)
	Finland	Non-ART	6,023,551	12,755	Ref	Ref	Ref
		ART	149,372	402	1.11 (1.00-1.23)	1.10 (0.99-1.22)	1.11 (1.00-1.23)
	Sweden	Non-ART	15,294,107	13,718	Ref	Ref	Ref
		ART	304,810	248	0.87 (0.77-0.99)	0.83 (0.73-0.94)	0.81 (0.72-0.93)
Ischemic heart disease	Combined	Non-ART	26,460,828	9,511	Ref	Ref	Ref
		ART	650,930	357	1.05 (0.94-1.17)	0.92 (0.82-1.03)	0.91 (0.81-1.01)
	Denmark	Non-ART	5,017,780	3,492	Ref	Ref	Ref
		ART	194,324	219	1.07 (0.93-1.24)	1.01 (0.88-1.17)	0.99 (0.86-1.14)
	Finland	Non-ART	6,078,809	2,220	Ref	Ref	Ref
		ART	150,859	71	0.93 (0.73-1.18)	0.92 (0.72-1.17)	0.93 (0.73-1.18)
	Sweden	Non-ART	15,364,240	3,349	Ref	Ref	Ref
		ART	305,746	67	0.88 (0.69-1.12)	0.80 (0.62-1.02)	0.78 (0.61-1.00)
Cerebrovascular disease	Combined	Non-ART	26,411,402	12,068	Ref	Ref	Ref
		ART	650,890	367	1.04 (0.94-1.16)	0.96 (0.87-1.07)	0.95 (0.86-1.06)
	Denmark	Non-ART	5,016,982	3,758	Ref	Ref	Ref
		ART	194,469	174	1.09 (0.93-1.28)	1.03 (0.88-1.21)	1.01 (0.86-1.18)
	Finland	Non-ART	6,072,393	3,429	Ref	Ref	Ref
		ART	150,733	113	1.12 (0.93-1.36)	1.10 (0.91-1.33)	1.11 (0.91-1.34)
	Sweden	Non-ART	15,352,028	4,881	Ref	Ref	Ref
		ART	305,688	80	0.78 (0.63-0.98)	0.76 (0.61-0.95)	0.75 (0.60-0.94)
Myocardial Infarction	Combined	Non-ART	26,492,194	3,229	Ref	Ref	Ref
		ART	652,257	95	0.95 (0.77-1.18)	0.84 (0.68-1.04)	0.83 (0.67-1.02)
	Denmark	Non-ART	5,032,534	801	Ref	Ref	Ref
		ART	195,170	41	1.04 (0.75-1.43)	0.94 (0.68-1.30)	0.91 (0.66-1.25)

© 2023 American Medical Association. All rights reserved.

	Finland	Non-ART	6,086,423	547	Ref	Ref	Ref
		ART	151,121	20	1.08 (0.68-1.71)	1.05 (0.66-1.65)	1.06 (0.67-1.68)
	Sweden	Non-ART	15,373,237	1,881	Ref	Ref	Ref
		ART	305,966	34	0.80 (0.57-1.13)	0.72 (0.51-1.02)	0.71 (0.50-1.00)
Stroke	Combined	Non-ART	24,456,115	9,305	Ref	Ref	Ref
		ART	651,255	282	1.04 (0.92-1.18)	0.96 (0.85-1.08)	0.95 (0.84-1.07)
	Denmark	Non-ART	5,021,130	2,927	Ref	Ref	Ref
		ART	194,638	136	1.12 (0.94-1.33)	1.05 (0.88-1.25)	1.02 (0.85-1.22)
	Finland	Non-ART	6,076,765	2,442	Ref	Ref	Ref
		ART	150,871	80	1.10 (0.88-1.38)	1.08 (0.86-1.36)	1.09 (0.87-1.37)
	Sweden	Non-ART	15,358,219	3,936	Ref	Ref	Ref
		ART	305,747	66	0.79 (0.62-1.01)	0.77 (0.60-0.98)	0.76 (0.59-0.97)
Cardiomyopathy	Combined	Non-ART	26,498,755	1,555	Ref	Ref	Ref
		ART	652,424	38	0.83 (0.60-1.15)	0.81 (0.59-1.13)	0.81 (0.58-1.13)
	Denmark	Non-ART	5,034,513	292	Ref	Ref	Ref
		ART	195,289	10	0.77 (0.40-1.46)	0.79 (0.41-1.50)	0.78 (0.41-1.50)
	Finland	Non-ART	6,085,582	592	Ref	Ref	Ref
		ART	151,128	15	0.83 (0.49-1.40)	0.82 (0.49-1.39)	0.82 (0.49-1.39)
	Sweden	Non-ART	15,378,659	671	Ref	Ref	Ref
		ART	306,007	13	0.88 (0.50-1.53)	0.84 (0.48-1.47)	0.83 (0.47-1.45)
Heart failure	Combined	Non-ART	26,495,920	2,378	Ref	Ref	Ref
		ART	652,425	58	0.79 (0.60-1.03)	0.72 (0.55-0.94)	0.71 (0.54-0.93)
	Denmark	Non-ART	5,033,568	561	Ref	Ref	Ref
		ART	195,251	23	0.86 (0.56-1.32)	0.80 (0.52-1.23)	0.78 (0.51-1.20)
	Finland	Non-ART	6,085,954	545	Ref	Ref	Ref
		ART	151,179	10	0.51 (0.27-0.95)	0.49 (0.26-0.92)	0.50 (0.26-0.93)
	Sweden	Non-ART	15,376,398	1,272	Ref	Ref	Ref
		ART	305,995	25	0.88 (0.58-1.31)	0.82 (0.54-1.22)	0.80 (0.54-1.20)
Pulmonary embolism	Combined	Non-ART	26,476,460	5,574	Ref	Ref	Ref
		ART	652,031	137	1.00 (0.84-1.19)	0.94 (0.79-1.12)	0.93 (0.78-1.11)
	Denmark	Non-ART	5,028,991	1,474	Ref	Ref	Ref
		ART	195,167	51	0.98 (0.74-1.31)	0.95 (0.72-1.27)	0.94 (0.70-1.25)
	Finland	Non-ART	6,081,601	1,397	Ref	Ref	Ref

© 2023 American Medical Association. All rights reserved.

		ART	150,999	47	1.29 (0.96-1.74)	1.27 (0.94-1.72)	1.28 (0.95-1.72)
	Sweden	Non-ART	15,365,869	2,703	Ref	Ref	Ref
		ART	305,865	39	0.76 (0.55-1.05)	0.73 (0.53-1.01)	0.72 (0.52-1.00)
Deep vein thrombosis	Combined	Non-ART	26,429,333	12,862	Ref	Ref	Ref
		ART	650,926	376	1.07 (0.97-1.19)	1.02 (0.92-1.13)	1.01 (0.91-1.12)
	Denmark	Non-ART	5,013,058	4,535	Ref	Ref	Ref
		ART	194,776	150	0.87 (0.74-1.03)	0.86 (0.73-1.02)	0.84 (0.71-1.00)
	Finland	Non-ART	6,055,546	5,790	Ref	Ref	Ref
		ART	150,368	176	1.19 (1.02-1.39)	1.19 (1.02-1.39)	1.20 (1.03-1.40)
	Sweden	Non-ART	15,360,729	2,537	Ref	Ref	Ref
		ART	305,781	50	1.17 (0.87-1.55)	1.09 (0.81-1.45)	1.07 (0.80-1.42)

[a] Adjusted for age, parity, polycystic ovarian syndrome, chronic hypertension, diabetes, year of start of follow-up, in addition to country (in the combined model).
[b] Adjusted for age, parity, polycystic ovarian syndrome, chronic hypertension, diabetes, year of start of follow-up, country (in the combined model), in addition to highest obtained educational level.

© 2023 American Medical Association. All rights reserved.

eTable 9 Risk of any cardiovascular disease according to conception by assisted reproductive technologies excluding pulmonary embolism and deep vein thrombosis from the outcome definition

Country	Exposure	N follow-up time in years	N events	Age and calendar year adjusted HR (95% CI)	Adjusted [a] HR (95% CI)
Combined	Non-ART	28,156,939	23,749	Ref	Ref
	ART	693,958	773	1.03 (0.96-1.11)	0.93 (0.87-1.00)
Denmark	Non-ART	5,043,171	7,829	Ref	Ref
	ART	194,484	394	1.08 (0.97-1.19)	1.02 (0.92-1.13)
Finland	Non-ART	6,649,034	6,807	Ref	Ref
	ART	158,165	201	0.98 (0.85-1.13)	0.97 (0.84-1.11)
Norway	Non-ART	545,576	263	Ref	Ref
	ART	24,586	15	0.83 (0.49-1.41)	0.77 (0.45-1.32)
Sweden	Non-ART	15,919,159	8,850	Ref	Ref
	ART	316,721	163	0.83 (0.71-0.98)	0.79 (0.67-0.92)

[a] Adjusted for age, parity, polycystic ovarian syndrome, diabetes, chronic hypertension, year of start of follow-up, in addition to country (in the combined model).

© 2023 American Medical Association. All rights reserved.

eTable 10 Risk of subgroups of cardiovascular disease according to conception by assisted reproductive technologies also including parous women at the start of follow-up

Outcome	Country	Exposure	N follow-up time in years	N events	Age and calendar year adjusted HR (95% CI)	Fully adjusted [a] HR (95% CI)
Any CVD	Combined	Non-ART	46,001,868	100,273	Ref	Ref
		ART	952,663	2,136	0.93 (0.89-0.97)	0.96 (0.91-1.00)
	Denmark	Non-ART	7,422,334	24,001	Ref	Ref
		ART	244,572	819	0.95 (0.89-1.02)	0.96 (0.89-1.03)
	Finland	Non-ART	9,590,403	24,710	Ref	Ref
		ART	204,536	612	1.04 (0.96-1.13)	1.06 (0.98-1.15)
	Norway	Non-ART	4,636,919	19,066	Ref	Ref
		ART	109,194	325	0.91 (0.82-1.02)	0.91 (0.81-1.01)
	Sweden	Non-ART	24,352,212	32,496	Ref	Ref
		ART	394,361	380	0.89 (0.80-0.98)	0.87 (0.78-0.96)
Ischemic heart disease	Combined	Non-ART	46,545,016	34,297	Ref	Ref
		ART	965,517	685	0.87 (0.80-0.94)	0.94 (0.87-1.01)
	Denmark	Non-ART	7,543,697	8,750	Ref	Ref
		ART	248,943	321	0.96 (0.86-1.08)	1.00 (0.89-1.12)
	Finland	Non-ART	9,738,506	5,983	Ref	Ref
		ART	208,327	128	0.90 (0.76-1.08)	0.94 (0.79-1.13)
	Norway	Non-ART	4,689,242	8,617	Ref	Ref
		ART	110,376	133	0.94 (0.79-1.12)	0.96 (0.81-1.15)
	Sweden	Non-ART	24,573,572	10,947	Ref	Ref
		ART	397,871	103	0.86 (0.71-1.05)	0.83 (0.68-1.02)
Myocardial Infarction	Combined	Non-ART	46,682,096	11,819	Ref	Ref
		ART	968,455	176	0.81 (0.70-0.95)	0.82 (0.70-0.95)
	Denmark	Non-ART	7,583,846	1,800	Ref	Ref
		ART	250,386	58	0.89 (0.68-1.15)	0.88 (0.68-1.15)
	Finland	Non-ART	9,764,216	1,618	Ref	Ref
		ART	208,977	32	0.86 (0.61-1.23)	0.88 (0.62-1.26)
	Norway	Non-ART	4,721,406	2,604	Ref	Ref
		ART	110,814	32	0.75 (0.53-1.06)	0.72 (0.51-1.03)

© 2023 American Medical Association. All rights reserved.

	Sweden	Non-ART	24,612,628	5,797	Ref	Ref
		ART	398,277	54	0.86 (0.65-1.12)	0.82 (0.62-1.07)
Cerebrovascular disease	Combined	Non-ART	46,529,988	30,306	Ref	Ref
		ART	964,888	612	0.91 (0.84-0.98)	0.92 (0.85-0.99)
	Denmark	Non-ART	7,546,164	6,837	Ref	Ref
		ART	248,816	239	0.99 (0.87-1.13)	0.97 (0.85-1.10)
	Finland	Non-ART	9,725,010	7,126	Ref	Ref
		ART	208,117	167	1.00 (0.86-1.17)	0.99 (0.85-1.16)
	Norway	Non-ART	4,706,917	5,037	Ref	Ref
		ART	110,509	80	0.87 (0.69-1.08)	0.85 (0.68-1.06)
	Sweden	Non-ART	24,551,898	11,306	Ref	Ref
		ART	397,446	126	0.83 (0.70-0.99)	0.82 (0.69-0.98)
Stroke	Combined	Non-ART	46,581,584	23,229	Ref	Ref
		ART	966,019	452	0.89 (0.81-0.98)	0.90 (0.82-0.99)
	Denmark	Non-ART	7,556,137	5,416	Ref	Ref
		ART	249,207	183	0.97 (0.84-1.13)	0.94 (0.81-1.10)
	Finland	Non-ART	9,736,756	5,338	Ref	Ref
		ART	208,388	118	0.97 (0.81-1.16)	0.98 (0.81-1.17)
	Norway	Non-ART	4,718,215	3,202	Ref	Ref
		ART	110,757	46	0.77 (0.58-1.04)	0.75 (0.56-1.01)
	Sweden	Non-ART	24,570,474	9,273	Ref	Ref
		ART	397,667	105	0.86 (0.71-1.05)	0.85 (0.70-1.03)
Cardiomyopathy	Combined	Non-ART	46,714,664	4,018	Ref	Ref
		ART	968,594	81	0.88 (0.70-1.09)	0.92 (0.73-1.15)
	Denmark	Non-ART	7,588,682	571	Ref	Ref
		ART	250,482	14	0.68 (0.40-1.17)	0.72 (0.42-1.23)
	Finland	Non-ART	9,764,329	1,158	Ref	Ref
		ART	208,958	23	0.85 (0.56-1.29)	0.84 (0.55-1.28)
	Norway	Non-ART	4,727,052	866	Ref	Ref
		ART	110,829	23	1.30 (0.86-1.98)	1.22 (0.80-1.86)
	Sweden	Non-ART	24,634,604	1,423	Ref	Ref
		ART	398,326	21	0.95 (0.61-1.46)	0.91 (0.59-1.42)
Heart failure	Combined	Non-ART	46,708,348	3,872	Ref	Ref
		ART	968,294	75	0.86 (0.68-1.08)	0.88 (0.69-1.10)
	Denmark	Non-ART	7,587,666	568	Ref	Ref
		ART	250,418	12	0.58 (0.33-1.05)	0.62 (0.35-1.10)
	Finland	Non-ART	9,763,309	1,119	Ref	Ref

© 2023 American Medical Association. All rights reserved.

		ART	208,871	22	0.83 (0.54-1.28)	0.83 (0.54-1.28)
	Norway	Non-ART	4,725,053	824	Ref	Ref
		ART	110,806	21	1.25 (0.81-1.93)	1.17 (0.75-1.81)
	Sweden	Non-ART	24,632,318	1,361	Ref	Ref
		ART	398,199	20	0.93 (0.60-1.47)	0.91 (0.58-1.43)
Pulmonary embolism	Combined	Non-ART	46,656,304	11,914	Ref	Ref
		ART	967,050	261	1.05 (0.95-1.19)	1.03 (0.91-1.17)
	Denmark	Non-ART	7,575,670	2,309	Ref	Ref
		ART	249,952	80	1.06 (0.85-1.33)	1.05 (0.83-1.31)
	Finland	Non-ART	9,756,047	2,574	Ref	Ref
		ART	208,679	72	1.20 (0.95-1.52)	1.20 (0.95-1.53)
	Norway	Non-ART	4,724,036	1,835	Ref	Ref
		ART	110,711	38	1.05 (0.76-1.45)	1.02 (0.74-1.41)
	Sweden	Non-ART	24,600,549	5,196	Ref	Ref
		ART	397,707	71	0.92 (0.73-1.17)	0.90 (0.71-1.15)
Deep vein thrombosis	Combined	Non-ART	46,492,380	26,126	Ref	Ref
		ART	963,116	634	1.01 (0.93-1.09)	1.06 (0.97-1.14)
	Denmark	Non-ART	7,532,433	7,334	Ref	Ref
		ART	248,683	219	0.89 (0.78-1.02)	0.91 (0.79-1.04)
	Finland	Non-ART	9,684,209	9,708	Ref	Ref
		ART	206,895	259	1.18 (1.04-1.33)	1.22 (1.08-1.38)
	Norway	Non-ART	4,712,268	3,924	Ref	Ref
		ART	110,463	83	1.00 (0.80-1.24)	0.99 (0.79-1.23)
	Sweden	Non-ART	24,563,469	5,160	Ref	Ref
		ART	397,075	73	1.13 (0.90-1.43)	1.10 (0.87-1.39)

[a] Adjusted for age, parity, polycystic ovarian syndrome, chronic hypertension, diabetes, year of start of follow-up in addition to country (in the combined model).

© 2023 American Medical Association. All rights reserved.

eTable 11 Stratified analysis of the risk of cardiovascular disease according to conception by assisted reproductive technologies by year of start of follow-up

Outcome	Year of start of follow-up	Exposure	N follow-up time in years	N events	Age and calendar year adjusted HR (95% CI)	Adjusted [a] HR (95% CI)	Adjusted [b] HR (95% CI)
Any CVD	1988-1997	Non-ART	14,525,361	22,435	Ref	Ref	Ref
		ART	148,374	320	0.92 (0.83-1.03)	0.94 (0.84-1.05)	0.92 (0.82-1.03)
	1998-2007	Non-ART	10,382,888	16,225	Ref	Ref	Ref
		ART	392,745	762	1.10 (1.02-1.18)	0.98 (0.91-1.05)	0.97 (0.90-1.05)
	2008-2017	Non-ART	3,139,035	4,025	Ref	Ref	Ref
		ART	150,560	221	1.06 (0.92-1.22)	1.02 (0.89-1.17)	1.01 (0.87-1.16)
Ischemic heart disease	1988-1997	Non-ART	14,638,827	6,041	Ref	Ref	Ref
		ART	149,700	105	0.83 (0.68-1.01)	0.83 (0.68-1.01)	0.83 (0.67-1.01)
	1998-2007	Non-ART	10,445,882	3,450	Ref	Ref	Ref
		ART	395,305	232	1.20 (1.05-1.38)	1.00 (0.87-1.14)	1.01 (0.87-1.16)
	2008-2017	Non-ART	3,145,155	528	Ref	Ref	Ref
		ART	150,881	33	0.87 (0.61-1.24)	0.82 (0.57-1.18)	0.81 (0.56-1.17)
Cerebrovascular disease	1988-1997	Non-ART	14,625,995	7,231	Ref	Ref	Ref
		ART	149,612	124	1.14 (0.95-1.37)	1.14 (0.95-1.37)	1.12 (0.93-1.36)
	1998-2007	Non-ART	10,439,131	4,598	Ref	Ref	Ref
		ART	395,338	212	1.03 (0.90-1.19)	0.92 (0.80-1.06)	0.93 (0.80-1.07)
	2008-2017	Non-ART	3,145,245	1,021	Ref	Ref	Ref
		ART	150,856	51	0.90 (0.68-1.20)	0.86 (0.64-1.15)	0.85 (0.63-1.13)
Myocardial Infarction	1988-1997	Non-ART	14,659,365	2,389	Ref	Ref	Ref
		ART	150,150	35	0.86 (0.61-1.20)	0.80 (0.57-1.13)	0.82 (0.58-1.16)
	1998-2007	Non-ART	10,457,377	895	Ref	Ref	Ref
		ART	396,167	56	1.02 (0.78-1.35)	0.86 (0.65-1.13)	0.88 (0.66-1.18)
	2008-2017	Non-ART	3,147,008	128	Ref	Ref	Ref
		ART	150,932	6	0.53 (0.23-1.22)	0.52 (0.22-1.18)	0.49 (0.21-1.14)
Stroke	1988-1997	Non-ART	14,635,493	5,752	Ref	Ref	Ref
		ART	149,792	90	1.03 (0.83-1.27)	1.03 (0.83-1.27)	1.01 (0.81-1.26)
	1998-2007	Non-ART	10,444,480	3,436	Ref	Ref	Ref
		ART	395,522	170	1.09 (0.93-1.27)	0.96 (0.82-1.12)	0.98 (0.83-1.15)

© 2023 American Medical Association. All rights reserved.

	2008-2017	Non-ART	3,145,911	704	Ref	Ref	Ref
		ART	150,877	38	0.94 (0.67-1.31)	0.89 (0.64-1.24)	0.87 (0.62-1.22)
Cardiomyopathy	1988-1997	Non-ART	14,665,334	947	Ref	Ref	Ref
		ART	150,244	8	0.60 (0.30-1.22)	0.60 (0.30-1.22)	0.53 (0.25-1.14)
	1998-2007	Non-ART	10,458,177	558	Ref	Ref	Ref
		ART	396,239	25	0.95 (0.63-1.44)	0.92 (0.61-1.39)	0.94 (0.62-1.44)
	2008-2017	Non-ART	3,146,809	213	Ref	Ref	Ref
		ART	150,924	10	0.82 (0.43-1.57)	0.84 (0.44-1.61)	0.81 (0.42-1.55)
Heart failure	1988-1997	Non-ART	14,663,146	1,566	Ref	Ref	Ref
		ART	150,195	21	0.82 (0.53-1.27)	0.77 (0.50-1.20)	0.80 (0.51-1.25)
	1998-2007	Non-ART	10,457,464	801	Ref	Ref	Ref
		ART	396,284	31	0.75 (0.52-1.08)	0.66 (0.46-0.95)	0.68 (0.47-0.99)
	2008-2017	Non-ART	3,146,862	192	Ref	Ref	Ref
		ART	150,929	13	1.24 (0.69-2.20)	1.25 (0.70-2.24)	1.26 (0.70-2.27)
Pulmonary embolism	1988-1997	Non-ART	14,651,391	3,014	Ref	Ref	Ref
		ART	150,139	22	0.63 (0.41-0.97)	0.62 (0.41-0.95)	0.62 (0.40-0.95)
	1998-2007	Non-ART	10,449,934	2,282	Ref	Ref	Ref
		ART	395,984	88	1.07 (0.86-1.33)	0.99 (0.80-1.23)	1.00 (0.80-1.25)
	2008-2017	Non-ART	3,145,813	680	Ref	Ref	Ref
		ART	150,893	32	1.15 (0.80-1.66)	1.08 (0.75-1.57)	1.09 (0.75-1.57)
Deep vein thrombosis	1988-1997	Non-ART	14,622,159	6,325	Ref	Ref	Ref
		ART	149,780	76	0.94 (0.74-1.18)	0.98 (0.78-1.24)	0.97 (0.76-1.22)
	1998-2007	Non-ART	10,430,809	5,960	Ref	Ref	Ref
		ART	395,288	228	1.06 (0.92-1.21)	0.96 (0.84-1.10)	0.92 (0.80-1.06)
	2008-2017	Non-ART	3,143,961	1,623	Ref	Ref	Ref
		ART	150,781	92	1.22 (0.98-1.52)	1.19 (0.96-1.48)	1.17 (0.94-1.45)

[a] Adjusted for age and parity, polycystic ovarian syndrome, chronic hypertension, diabetes, year of start of follow-up in addition to country in the pooled model.
[b] Adjusted for age and parity, polycystic ovarian syndrome, chronic hypertension, diabetes, year of start of follow-up, country in the pooled model, in addition to tobacco use and body-mass index.

© 2023 American Medical Association. All rights reserved.
