# Supplementary material for: Maternal Risk of Cardiovascular Disease After Use of Assisted Reproductive Technologies
Source: JAMA Cardiol. 2023 Aug 9;8(9):837–45. doi: 10.1001/jamacardio.2023.2324 (PMC10413220; doi:10.1001/jamacardio.2023.2324)
Supplement: Supplement 2. — Data Sharing Statement [file jamacardiol-e232324-s002.pdf]

Data Sharing Statement

Magnus. Maternal Risk of Cardiovascular Disease After Use of Assisted Reproductive Technologies. *JAMA Cardiol*. Published August 09, 2023. doi:10.1001/jamacardio.2023.2324

Data
Data available: No

Additional Information
Explanation for why data not available: Individual level data will can't be made available for privacy reason according to national laws.
